# Short-term exposure sequences and anxiety symptoms: a time series clustering of smartphone-based mobility trajectories

**DOI:** 10.1186/s12942-023-00348-1

**Published:** 2023-10-10

**Authors:** Yuliang Lan, Marco Helbich

**Affiliations:** Department of Human Geography and Spatial Planning, Faculty of Geosciences, Utrecht University, Princetonlaan 8a, 3584 BC Utrecht, The Netherlands

**Keywords:** Exposure sequences, Green space, Air pollution, Noise, Daily mobility, Global positioning system, Time series

## Abstract

**Background:**

Short-term environmental exposures, including green space, air pollution, and noise, have been suggested to affect health. However, the evidence is limited to aggregated exposure estimates which do not allow the capture of daily spatiotemporal exposure sequences. We aimed to (1) determine individuals’ sequential exposure patterns along their daily mobility paths and (2) examine whether and to what extent these exposure patterns were associated with anxiety symptoms.

**Methods:**

We cross-sectionally tracked 141 participants aged 18–65 using their global positioning system (GPS) enabled smartphones for up to 7 days in the Netherlands. We estimated their location-dependent exposures for green space, fine particulate matter, and noise along their moving trajectories at 10-min intervals. The resulting time-resolved exposure sequences were then partitioned using multivariate time series clustering with dynamic time warping as the similarity measure. Respondents’ anxiety symptoms were assessed with the Generalized Anxiety Disorders-7 questionnaire. We fitted linear regressions to assess the associations between sequential exposure patterns and anxiety symptoms.

**Results:**

We found four distinctive daily sequential exposure patterns across the participants. Exposure patterns differed in terms of exposure levels and daily variations. Regression results revealed that participants with a “moderately health-threatening” exposure pattern were significantly associated with fewer anxiety symptoms than participants with a “strongly health-threatening” exposure pattern.

**Conclusions:**

Our findings support that environmental exposures’ daily sequence and short-term magnitudes may be associated with mental health. We urge more time-resolved mobility-based assessments in future analyses of environmental health effects in daily life.

**Supplementary Information:**

The online version contains supplementary material available at 10.1186/s12942-023-00348-1.

## Background

Evidence steadily consolidates that accounting for dynamic environmental exposures is likely more accurate than residence-based approaches, not factoring in exposures incurred during people’s day-to-day mobility [1, 2]. Only a few studies have relied on global positioning system (GPS)-enabled sensing technologies to measure people’s exposures at out-of-home activity locations and along their mobility paths [3–6]. Given the high spatiotemporal granularity of tracking data, location-dependent exposure sequences can be derived [7]. However, to date, no studies have appeared to have done so.

State-of-the-art mobility-based exposure assessments focus primarily on the health effects of aggregated exposures (e.g., daily or weekly exposure averages per person) [8–12]. However, such aggregation might average out short-term spatiotemporal variabilities in exposures, which likely have different health effects than exposure averages [13]. A US study supports this speculation showing that asthma symptoms could be associated with 1-h maximum exposure to ambient particulate matter (PM_10_); however, null associations were found using 24-h average PM_10_ exposures [14]. Another study also observed a similar phenomenon in which the daily mean PM_2.5_ concentrations were thought to underestimate the cardiovascular mortality burden due to hourly PM_2.5_ variations being ignored [15]. Therefore, solely using mean values of spatiotemporal exposures along individuals’ daily mobility paths to characterize their exposures is possibly an oversimplified approach that overlooks temporal variations.

Based on GPS tracking data, the measurement of short-term daily exposure sequences allows to consider both magnitude and temporal fluctuations of exposures [2]. Further, exposures captured at different daily time points may have differential impacts on health [16–18]. Thus, there is a significant need for dynamic exposure conceptualizations that incorporate spatiotemporal exposure sequences along people’s daily mobility paths [19].

Our exploratory study had two aims for bridging this research gap. First, to characterize individuals’ daily sequential exposure patterns of green space, noise, and PM_2.5_ along their mobility paths based on time series clustering. Second, using anxiety symptoms as an example, we aim to examine how different daily sequential exposure patterns were associated mental health.

## Methods

### Mobile phone-based GPS data collection

We acquired GPS data from the NEEDS (‘Dynamic Urban Environmental Exposures on Depression and Suicide’) study collected between September and November 2018 in the Netherlands [20]. Survey respondents aged 18 to 65 (*N* = 11,505) who agreed to be re-contacted were invited via email to download our Android “Your Living Environment” mobile phone application to collect movement data. We sent 8869 invitation emails within 2 days of survey completion. To increase participation in the mobile phone-based GPS data collection, we raffled off 400 gift vouchers valued at €22 each. In total, 821 participants downloaded the app (i.e., 7.1% of the survey respondents), and 629 permitted tracking and recording.

The locational information was recorded every 20 s for subjects in motion. The recording frequency decreased to 1 min if the mobile phone showed no movement (i.e., displacement of the phone < 20 m) after 30 min. If the mobile phone was stationary for over 1 h, the location was recorded every 2 min to conserve battery power. After a cumulative total of 7 days of data collection, the app stopped recording. Data, initially stored locally, were then uploaded daily to a secure server at Utrecht University.

### Preprocessing of the GPS data

The GPS data were cleaned following established practice [3, 4]. Data cleaning entailed the removal of inaccurate GPS points and participants whose records were not representative of a typical week (e.g., leaving the country) (Additional file 1: Table S1). This cleaning process preserved 419 participants with 685,971 GPS points provided.

Recording a sufficient quantity of GPS locations was necessary to derive meaningful tracks capturing people’s daily movement and their related spatiotemporal environmental exposures. However, locational sampling coverage was likely to be sparse due to technological constraints (e.g., signal loss in urban areas), battery depletion, or phones temporarily being turned off. To evaluate the quality of the daily GPS data, for each participant, we enumerated how many daytime hours (6:00 am to 10:00 pm, 16 h in total) included at least one recorded location (Additional file 1: Table S2). We assumed people remained home for 8 nighttime hours, from 10:00 pm to 6:00 am. According to Additional file 1: Table S2, only a limited number of participants had complete GPS records for all 7 days (i.e., every day included GPS-based data points for all 16 daytime hours).

Given the limited number of complete GPS records, our study defined days with 4 h or less of missing GPS data (i.e., a minimum of 12 daytime hours with GPS records) as valid days providing sufficient daily locational data. Those with at least 4 valid days were eligible for inclusion to represent participants’ typical weekly mobility. Considering the possibility of different travel behaviors on weekends versus weekdays [21], we included participants with a minimum of 3 valid weekdays and 1 valid weekend day. In cases in which participants had more valid weekdays or weekend days than the minimum required, we randomly selected 3 weekdays and 1 weekend day to ensure each participant had the same number of days of data included. Our final sample included 141 participants.

### Environmental exposure data

#### Green space

We used the Normalized Difference Vegetation Index (NDVI) as our green space metric [22]. The NDVI was derived from Landsat 8 imagery with a cloud cover of < 40% for 2018 obtained through Google Earth Engine [23]. Scenes had a resolution of 30 m × 30 m. We only included atmospherically corrected images collected between May and September, when vegetation is at its greenest. We removed pixels with a cloud score of > 25 before determining the median NDVI per pixel. Negative NDVI values were masked to avoid distortion. Higher positive NDVI values represented higher levels of vegetation.

#### Noise

Average day–night–evening (Lden [dB]) noise levels were calculated according to the Standard Model Instrumentation for Noise Assessments (STAMINA). This model considered noise sources from roads, rails, air traffic, industry, and wind turbines for 2016 [24]. The noise data resolution varied depending on the distance between the noise source and the observation point, with resolution values increasing from 10 to 80 m based on increasing distance [25].

#### Air pollution

We acquired yearly averaged concentrations of fine (≤ 2.5 µm) particulate matter (PM_2.5_) (µgm^−3^) from a nationwide land-use regression model [26]. The model regressed monitored PM_2.5_ concentrations on land use, traffic infrastructure, traffic intensity, and population density for 2009. The calibrated model was then used to predict PM_2.5_ concentrations at unsampled locations. We resampled the data from 5 to 25 m to reduce program run time. The air pollution data set we used showed that air pollution values had remained stable for nearly a decade prior to our survey and were, thus, applicable for use in our analysis [27].

### Determining space-time exposure series

**Fig. 1:**
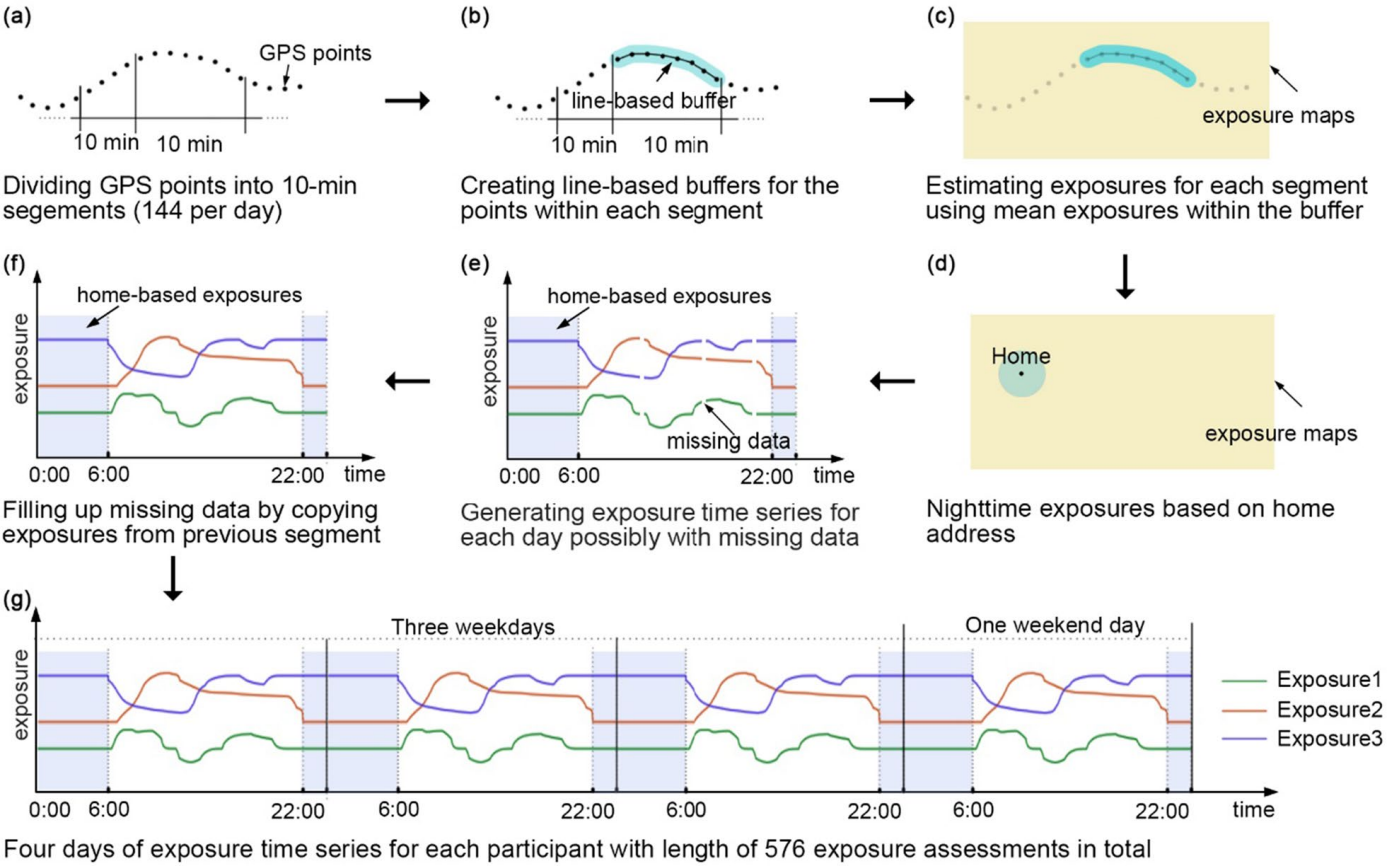
Assessment of exposure time series based on the GPS tracking data

Following a previous GPS study [28], environmental exposures were assessed every 10 min along a respondent’s moving trajectory, resulting in 144 daily 10-min segments (Fig. 1a). We created line-based buffers of 100 m for each trajectory segment (Fig. 1b), a typical buffer width used elsewhere [3, 4, 29]. Mean NDVI, noise, and PM_2.5_ exposure estimates were determined per segment within a buffer (Fig. 1c). The 48 nighttime exposure segments (10:00 pm to 6:00 am, 8 h) were assessed using home-based buffers (i.e., 100 m) (Fig. 1d). Home addresses were geocoded by matching the population register with the cadaster. For some of the daytime segments without GPS records (Fig. 1e), interpolation was conducted by copying the exposures from the previous segment (Fig. 1f), assuming people were static during this period of missing data. Consequently, for each participant, we received three (i.e., NDVI, noise, and PM_2.5_) time series of environmental exposures with a length of 576 short-term exposure assessments (144 × 4 days) (Fig. 1g). All participants’ exposures for their 3 weekdays were placed before their weekend exposures to ensure consistent weekday-weekend ordering of data. For our sensitivity analyses, we repeated the procedure using 50 m buffers and 30-min time windows (48 segments per day). The latter resulted in exposure series with a length of 192 exposure assessments (48 × 4 days).

### Multivariate time series clustering

Based on the space-time exposure series obtained from “Determining space-time exposure series” section, we employed multivariate time series clustering with constrained dynamic time warping as a distance metric to determine environmental exposure patterns [30]. Due to differences in exposure units, we normalized each exposure time series using *z*-scores. To achieve clustering results with optimal performance, we tested a varying number of clusters (*k*) from 2 to 8 (Additional file 1: Fig. S1) and the window size of constraints (i.e., the maximum amount of warping) from 0.5 to 2.5 h (Additional file 1: Fig. S2). For each combination of the two parameters, we repeated the clustering procedure 100 times as the algorithm required a random starting value, which could potentially affect the partition results [31]. We used the Davies–Bouldin index to identify an optimal number of clusters (*k*) and the window size of constraints [32]. Smaller index values indicate better clustering performance [33]. The results with the smallest DB value were used. Separate time series clustering runs were conducted for different buffer sizes (50 and 100 m) and time windows (10 and 30 min). We visualized the diurnal multi-exposure patterns of each cluster using hourly line charts, where the exposure values of each hour referred to the averages of the corresponding hour over 4-day spans.

### Statistical analyses

We developed regression models to test the associations between anxiety symptoms and sequential exposure patterns as a pilot study. Anxiety symptoms over the last 2 weeks were measured during the survey using the Generalized Anxiety Disorder-7 (GAD-7) questionnaire [34]. Participants reported on seven questions, such as “feeling nervous, anxious or on edge” or “not being able to stop or control worrying.“ Response options to each question ranged from 0 (“Not at all”) to 3 (“Nearly every day”). We summed the individual item scores resulting in GAD-7 scores ranging from 0 to 21. A higher overall score indicated more severe anxiety symptoms. The Cronbach’s alpha was 0.90 between individual items.

Models were adjusted for age, sex, income (quintiles treated continuously), employment status (employed, unemployed), marital status (married, unmarried), and educational background. Educational background was scored as high (undergraduate or graduate university education), medium (upper secondary education), and low (up to lower secondary education). We fitted separate models for different buffer sizes (50 and 100 m) and time windows (10 and 30 min) for sensitivity tests. All analyses were conducted using R software, version 4.1.3 [35].

## Results

### Descriptive statistics

Table 1 shows the descriptive statistics for our sample. The average GAD-7 score was 4.10, with a standard deviation (SD) of ± 4.34. The mean age of the participants was 43.66, and the majority were employed (72.3%). The gender and marital status divisions were approximately equal. Most respondents were middle or highly educated with high or very high incomes. The demographic and socioeconomic characteristics of the analytical sample closely mirrored those before the preprocessing of the GPS data (Additional file 1: Table S3).

**Table 1:** Summary statistics of the sample

Variables	Category	Analytical sample (*N* = 141)
GAD-7 score	Mean (SD)	4.099 (4.341)
Age	Mean (SD)	43.660 (14.047)
Sex	Male [*N* (%)]	73 (51.8%)
	Female [*N* (%)]	68 (48.2%)
Employment	Employed [*N* (%)]	102 (72.3%)
	Unemployed [*N* (%)]	39 (27.7%)
Income	Mean (SD)	3.574 (1.321)
Marital status	Married [*N* (%)]	78 (55.3%)
	Unmarried [*N* (%)]	63 (44.7%)
Education	Low [*N* (%)]	14 (9.9%)
	Mid [*N* (%)]	61 (43.3%)
	High [*N* (%)]	66 (46.8%)

### Sequential environmental exposure patterns

**Fig. 2:**
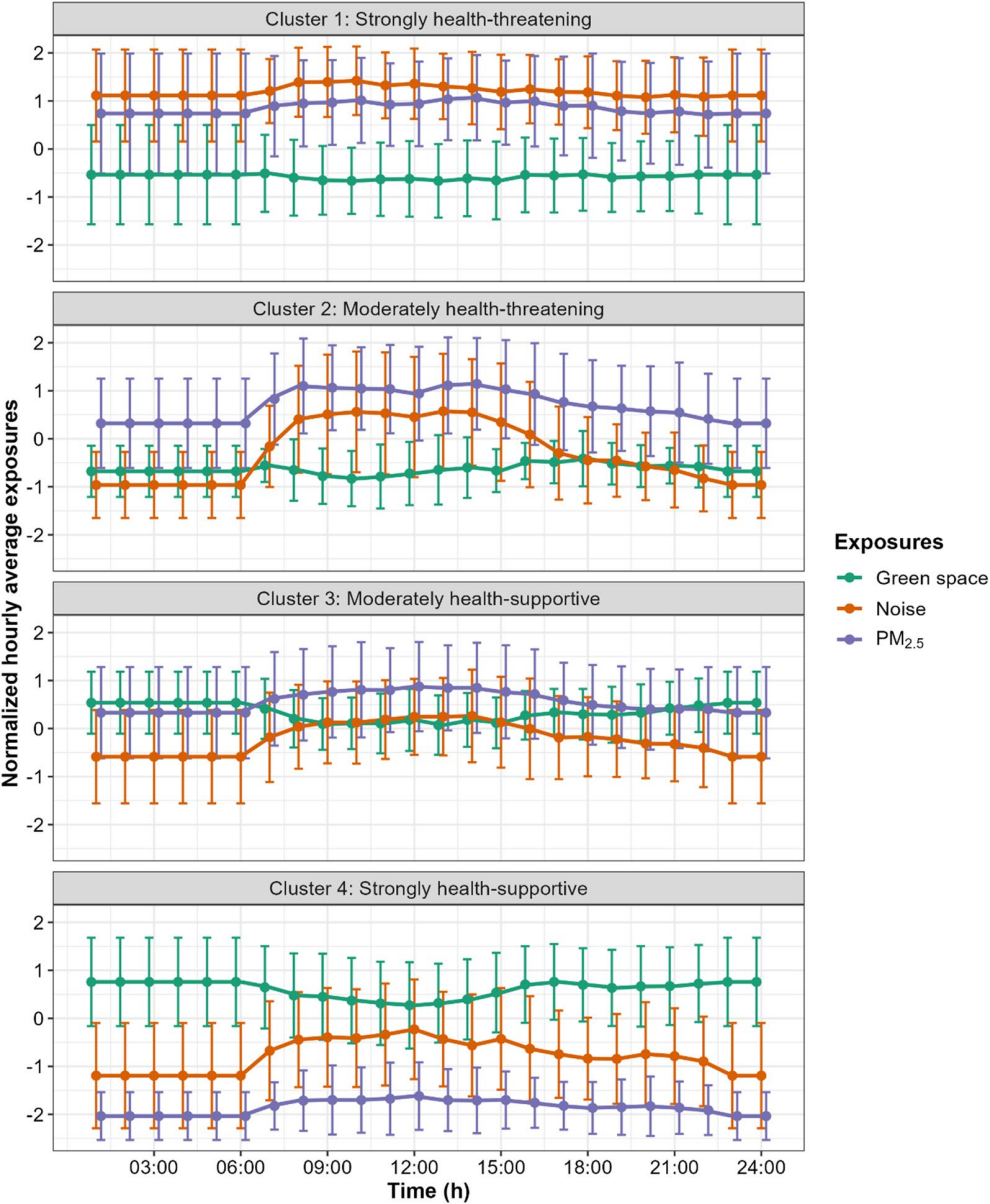
Sequential environmental exposure patterns of the four clusters based on 100 m buffers and 10-min time windows. Exposure means and related SDs are shown for each exposure in each cluster. The data partition was based on multivariate time series clustering with constrained dynamic time warping

The Davies–Bouldin index suggested that four clusters were optimal for the multivariate time series clustering (Additional file 1: Fig. S1). Figure 2 shows each cluster’s sequential environmental exposure patterns using buffers of 100 m and time windows of 10 min. Participants in each cluster were exposed to different ambient environments, which revealed distinctive daily exposure variations. Based on the exposure characteristics, we labeled the clusters as “strongly health-threatening,” “moderately health-threatening,” “moderately health-supportive,” and “strongly health-supportive.” Those in the “strongly health-threatening” cluster (*N* = 38) were constantly exposed to high noise and high PM_2.5_ concentrations in conjunction with lesser green space exposure. Participants in the “moderately health-threatening” cluster (*N* = 23) constantly experienced lesser green space exposure and relatively low noise at night but striking noise increases during the daytime, with PM_2.5_ exposure being relatively high at night and increasing further during the day. Participants allocated to the “moderately health-supportive” cluster (*N* = 40) were exposed to relatively high amounts of green space with a decrease in green space exposure during the daytime and moderately high noise and PM_2.5_ levels with a slight increase during the daytime. The “strongly health-supportive” cluster (*N* = 40) included participants exposed to high amounts of green space but with a decrease during the daytime, along with low noise and PM_2.5_ exposures at nighttime but which increased during the day.

### Associations between anxiety symptoms and exposure patterns

**Fig. 3:**
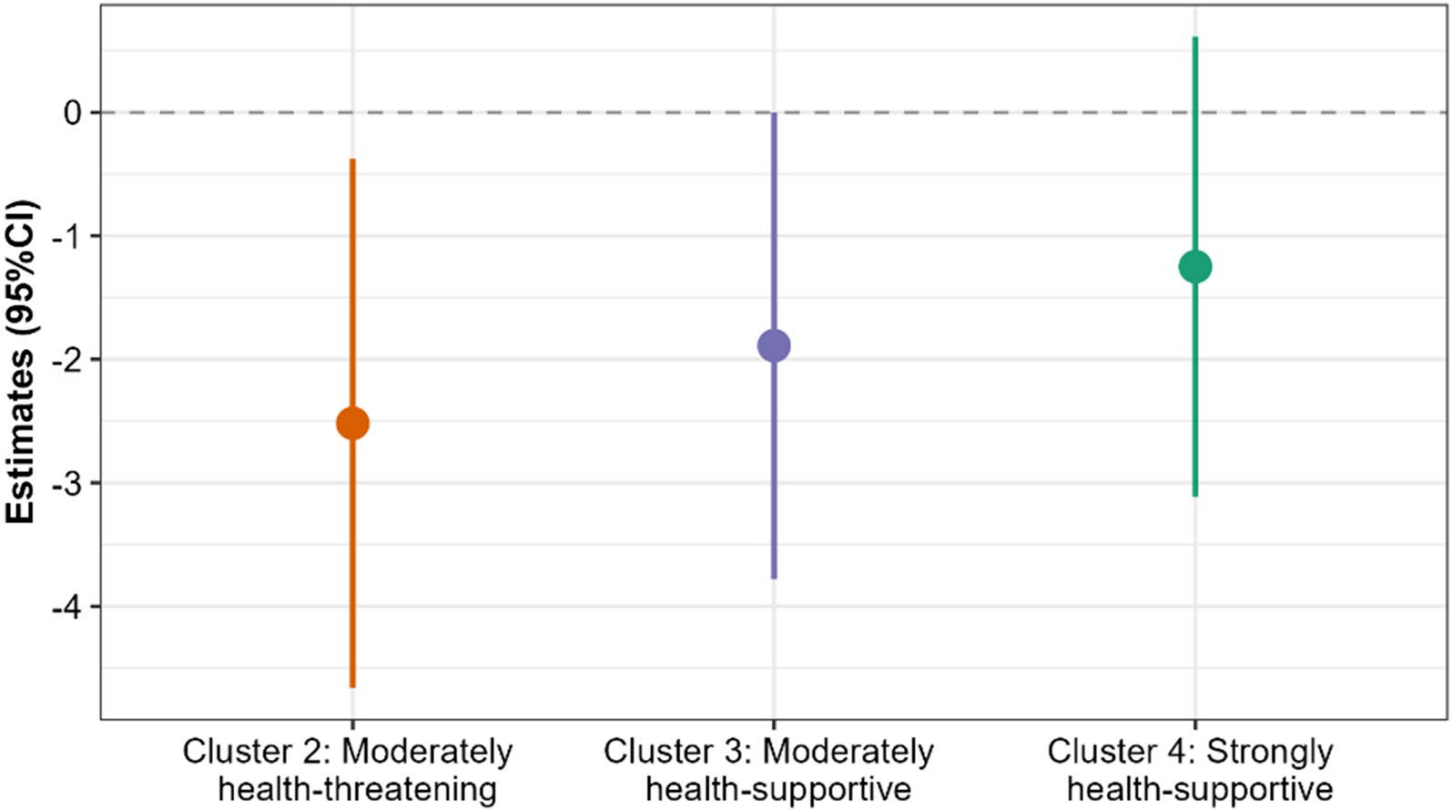
Regression coefficients and 95% CIs for the associations between the GAD-7 scores and the exposure patterns. Cluster 1 (“strongly health-threatening”) served as the reference category. The model was adjusted for age, sex, income, employment status, marital status, and educational background. Exposures were based on 100 m buffers and 10 min time windows

Figure 3 illustrates the regression results between anxiety symptoms and the exposure patterns for the 100 m buffers and 10-min time windows. The signs of the coefficients indicated that, on average, participants in the “moderately health-threatening,“ “moderately health-supportive,“ and “strongly health-supportive” clusters tended to be associated with lower GAD-7 scores than participants in the reference cluster (i.e., “strongly health-threatening”). However, only the “moderately health-threatening” cluster reached statistical significance (*p* < 0.05); the negative coefficient of the “moderately health-supportive” cluster was marginally significant (Additional file 1: Table S4). Unexpectedly, the negative coefficient of the “strongly health-supportive” cluster was insignificant.

### Sensitivity analyses

Our sensitivity analyses revealed that the sequential environmental exposure patterns of the 50 m buffers (Additional file 1: Fig. S3) and the 30-min time windows (Additional file 1: Figs. S4 and S5) resulted in four clusters largely congruent with our main results. Only minor differences were noticeable. For example, for the 50 m buffers (Additional file 1: Fig. S3), the “moderately health-supportive” cluster had lower overall PM_2.5_ exposure, and the “moderately health-threatening” cluster had higher nighttime noise exposure compared with the 100 m buffers (Fig. 2).

The regression results were broadly consistent across the 50 and 100 m buffers (Additional file 1: Fig. S6). In the model with 50 m buffers, the “moderately health-supportive” cluster was statistically significant (*p* < 0.05) instead of the “moderately health-threatening” cluster. Sensitivity tests with the 30-min time windows resulted in similar negative, but non-significant, coefficients of the three clusters (i.e., “moderately health-threatening,“ “moderately health-supportive,“ and “strongly health-supportive”) compared to the “strongly health-threatening” cluster (Additional file 1: Fig. S6).

## Discussion

### Main findings

The few available mobility-based studies on environmental-health associations typically have assessed aggregated exposures over time (e.g., daily or weekly). Our study broke new ground by proposing a novel methodology to determine temporally disaggregated sequential exposure patterns along individuals’ GPS-tracked mobility paths. We identified four sequential exposure patterns among our participants based on the magnitude of the experienced environmental exposures (i.e., green space, noise, and PM_2.5_) along the daily moving trajectories. Participants in different clusters differed in their overall exposure levels and daily exposure variation, which traditional exposure averages have difficulty capturing. The regression results of our pilot study revealed that the varying sequential exposure patterns were differently associated with anxiety symptoms. Particularly, those participants with a “moderately health-threatening” exposure pattern were associated with significantly fewer anxiety symptoms than participants with a “strongly health-threatening” exposure pattern. However, the associations between anxiety symptoms and the sequential exposure patterns did not pass all sensitivity tests, especially when using 30-min exposure time windows.

### Characterizing sequential exposure patterns using time series clustering approach

Our study demonstrated the usefulness of time series clustering to determine individuals’ sequential spatiotemporal exposure patterns. This dynamic exposure conceptualization considers the co-occurrence of disaggregated exposures over space-time. Characterizing exposures in this manner will likely provide exposure summarizations that differ from past studies’ aggregated exposure assessments [36]. Unlike traditional sequence analysis, constrained to categorical data [37, 38], time series clustering enables continuous exposure metrics, which are more desirable because they require neither exposure categorization nor arbitrarily defined distance metrics between different categories [28].

The temporal granularity (i.e., the time interval of the exposure windows) is an important parameter that could affect the clustering results [39]. A too-fine temporal resolution may introduce much, probably less meaningful, fluctuation, while a too-coarse temporal resolution may average the exposure variations [40]. We observed significant anxiety-related associations using 10-min exposure windows, but these results were not replicated when using 30-min time windows. Our study uncovered granularity-related temporal uncertainties that parallel other well-established issues with defining health-influencing geographic contexts [41]. Future studies are encouraged to carefully specify both the temporal and spatial exposure windows, which may depend on whether the analyses rely on short-term exposures along people’s daily mobility or long-term over their life course.

### Sequential exposure patterns and anxiety symptoms

Since our pilot study is the first we are aware of that deals with daily sequential exposure patterns, we embed our findings in the broader debate over the impact of the environment on mental health [42–44]. Our regression results suggested that participants exposed to a severely compromised ambient environment (i.e., “strongly health-threatening” cluster) tended to be associated with more anxiety symptoms. This finding is in line with previous studies that had reported positive associations between anxiety and exposure to noise [45, 46] and PM_2.5_ [8, 45], especially when the exposure levels were high [3, 47]. Moreover, we also found that participants with a “moderately health-threatening” exposure pattern were associated with significantly fewer anxiety symptoms than those with a “strongly health-threatening” exposure pattern. The most apparent difference between the two clusters was that participants in the “moderately health-threatening” cluster had significantly less nighttime noise exposure. This lesser exposure may have significantly contributed to their relative paucity of anxiety symptoms, as nighttime noise has been recognized as a more pronounced risk factor than daytime noise [18, 48].

Participants in the “moderately health-supportive” and “strongly health-supportive” clusters had moderate or high green space exposure. However, their negative coefficients did not reach statistical significance, implying that participants exposed to more green space did not necessarily to be associated with significantly fewer anxiety symptoms. A possible explanation is that, despite high overall green space exposures, these participants tended to experience significantly less green space during the daytime when they were most active. Another possible explanation is that individuals with more anxiety symptoms may spend more time in green and quiet places for self-regulation purposes. The insignificant result differs from previous studies reporting significant negative associations between green space exposure and anxiety [49–52]. However, in line with our results, others have also reported null associations [53–55].

Our study indicated that the exposure magnitude and daily variations of multiple exposures might jointly affect anxiety symptoms, although rarely previously recognized. We speculate that individuals’ daily mood fluctuations are influenced by their biological clocks [56, 57] and their daily dynamic exposures [58]. Supported by prior studies [59, 60], these fluctuations in immediate moods can shape individuals’ mental well-being over time. This rationale may explain why daily sequential exposure patterns could be related to people’s mental health status.

### Strengths and limitations

This mobility-based study went beyond exposure averages to daily sequential exposure patterns. Such sequential exposure patterns consider both the magnitude and variation of exposures, which is impossible with traditional aggregation-based approaches. Additional file 1: Figures S7 and S8 show the distinction between our time-series approach and the aggregation-based approach when preprocessing mobility-based exposure data. The multivariate time series clustering enabled us to simultaneously include multiple exposures over space and time. This dynamic exposure assessment took advantage of the spatiotemporal exposure measures obtained from GPS tracking and has the potential to contribute new insights into exposure-health associations. Finally, while many previous GPS-based studies had study domains limited to a single city [9, 61–63], we tested our approach based on a nationwide sample from the Netherlands, providing data from diverse environmental settings.

The study also had several limitations. First, the PM2.5 and noise data, derived for 2009 and 2016, may not entirely reflect the air pollution and noise levels in 2018 when the GPS data were collected. Second, the exposure assessment was based on exposure maps representing annual averages, which neglect temporal exposure fluctuations and, in turn, may lead to some exposure misclassification, especially for air pollution, which faces diurnal variations [64]. To realize the full advantage of our time series approach, spatiotemporally resolved exposure maps (e.g., on an hourly level) should be incorporated in the future to explore the full potential of our approach. Third, our pilot study only measured anxiety symptoms at baseline. Our analysis was conducted under the assumption that individuals’ anxiety symptoms and their daily sequential exposure patterns remained stable throughout our tracking period of 1 week. It was reported that most people with high GAD-7 scores have chronic symptoms for a month or more [34]. Elsewhere, it was also shown that individuals’ day-to-day mobility patterns are relatively stable [65]. Our cross-sectional design limited our ability to establish causal relationships. We advice future studies to record mental health responses repeatedly using geographically-explicit ecological momentary assessments [66]. Fourth, as with most GPS-based studies [11, 61], we cannot rule out individuals’ selective daily mobility bias [67, 68]. Fifth, our sample was not representative of the Dutch population. Most included participants were highly educated with relatively higher incomes, which would challenge the generalization of the results, but this study introduced a methodological innovation. Finally, obtaining enough hourly GPS tracks for each participant was challenging and resulted in a relatively small final sample. We also cannot guarantee that our sample has represented all possible exposure patterns and using a different sample could yield slightly different sequential exposure patterns. Thus, future studies with more extensive, high-quality samples are needed to examine how exposure sequences are possibly associated with health outcomes.

## Conclusions

We proposed a novel GPS-based methodology to determine individuals’ sequential environmental exposure patterns along their daily mobility. Our findings support the previously neglected notion that people’s daily sequential exposure patterns may play a role in mental health. Our data on GPS-tracked Dutch adults showed four distinctive daily sequential exposure patterns based on people’s daily mobility paths. Each pattern was composed of multiple exposures, which differed in magnitudes and daily exposure variations. The regression analyses provided suggestive evidence that some daily exposure patterns were associated with anxiety symptoms. We advise future studies using more extensive tracking data to replicate our approach using time-resolved exposure assessments to uncover spatiotemporally based environmental impacts on mental health.

### Supplementary Information

**Additional file 1: Table S1.** GPS data cleaning. **Table S2.** The number of participants with different GPS data quality. **Table S3.** Sample characteristics before and after GPS cleaning process. **Table S4.** Associations between the GAD-7 scores and the exposure patterns. Exposures were based on 100 m buffers and 10 min time window. The model was adjusted for age, sex, income, employment status, marital status, and educational background. **Figure S1.** Davies Bouldin index for different numbers of clusters and across various model settings (i.e., time windows and buffer sizes). **Figure S2.** Davies Bouldin index for different window size of constraints and across various model settings (i.e., time windows and buffer sizes). **Figure S3.** Sequential environmental exposure patterns of the four clusters based on 50 m buffers and 10-min time windows. The data partition was conducted with multivariate time series clustering and constrained dynamic time warping. **Figure S4.** Sequential environmental exposure patterns of the four clusters based on 100 m buffers and 30-min time windows. The data partition was conducted with multivariate time series clustering and constrained dynamic time warping. **Figure S5.** Sequential environmental exposure patterns of the four clusters based on 50 m buffers and 30-min time windows. The data partition was conducted with multivariate time series clustering and constrained dynamic time warping. **Figure S6.** Regression coefficients for the associations between the GAD-7 scores and the exposure patterns across different buffers (50 m and 100 m) and time windows (10 min and 30 min). Cluster 1 (“strongly health-threatening”) served as the reference category. The model was adjusted for age, sex, income, employment status, marital status, and educational background. **Figure S7.** Preprocessing of mobility-based exposure data using aggregation-based approach. **Figure S8.** Preprocessing of mobility-based exposure data in our study using the time-series approach. Individuals of the same color were assigned in the same cluster.

## Data Availability

The data used in this analysis cannot be shared with third parties due to privacy restrictions.
