# Supplementary material for: Short-term exposure sequences and anxiety symptoms: a time series clustering of smartphone-based mobility trajectories
Source: Int J Health Geogr. 2023 Oct 10;22:27. doi: 10.1186/s12942-023-00348-1 (PMC10563352; doi:10.1186/s12942-023-00348-1)
Supplement: Supplementary file 1 — Additional file 1: Table S1. GPS data cleaning. Table S2. The number of participants with different GPS data quality. Table S3. Sample characteristics before and after GPS cleaning process. Table S4. Associations between the GAD-7 scores and the exposure patterns. Exposures were based on 100 m buffers and 10 min time window. The model was adjusted for age, sex, income, employment status, marital status, and educational background. Figure S1. Davies Bouldin index for different numbers of clusters and across various model settings (i.e., time windows and buffer sizes). Figure S2. Davies Bouldin index for different window size of constraints and across various model settings (i.e., time windows and buffer sizes). Figure S3. Sequential environmental exposure patterns of the four clusters based on 50 m buffers and 10-min time windows. The data partition was conducted with multivariate time series clustering and constrained dynamic time warping. Figure S4. Sequential environmental exposure patterns of the four clusters based on 100 m buffers and 30-min time windows. The data partition was conducted with multivariate time series clustering and constrained dynamic time warping. Figure S5. Sequential environmental exposure patterns of the four clusters based on 50 m buffers and 30-min time windows. The data partition was conducted with multivariate time series clustering and constrained dynamic time warping. Figure S6. Regression coefficients for the associations between the GAD-7 scores and the exposure patterns across different buffers (50 m and 100 m) and time windows (10 min and 30 min). Cluster 1 (“strongly health-threatening”) served as the reference category. The model was adjusted for age, sex, income, employment status, marital status, and educational background. Figure S7. Preprocessing of mobility-based exposure data using aggregation-based approach. Figure S8. Preprocessing of mobility-based exposure data in our study using the time-series approach. Individuals o [file 12942_2023_348_MOESM1_ESM.docx]

**Additional file 1**

Table S1: GPS data cleaning.

|  | Number of participants | Number of GPS points |
| --- | --- | --- |
| Number of times the app was downloaded | 821 |  |
| Agreed to permissions and provided GPS data | 629 | 1,123,409 |
| Removal of participants with fewer than 2.5 times the median absolute deviation of GPS points | 37 | 361 |
| Removal of GPS points with a speed >200 km/hr | 0 | 14,002 |
| Removal of GPS points within 100 m of German/Belgian border | 0 | 878 |
| Removal of participants that left the Netherlands during data collection | 173 | 384,951 |
| Removal of GPS points >50 m from travel network | 0 | 37,246 |
| Final sample | 419 | 685971 |

Table S2: The number of participants with different GPS data quality.

| Number of hours with valid GPS records during 6:00-22:00 (16 hr in total) | Number of days | | | | | | |
| --- | --- | --- | --- | --- | --- | --- | --- |
|  | 1 day | 2 days | 3 days | 4 days | 5 days | 6 days | 7 days |
| 8 hours | 365 | 328 | 288 | 263 | 229 | 196 | 148 |
| 10 hours | 325 | 274 | 238 | 207 | 176 | 143 | 101 |
| 12 hours | 270 | 220 | 180 | 153 | 128 | 88 | 46 |
| 14 hours | 206 | 148 | 112 | 89 | 69 | 43 | 20 |
| 16 hours | 118 | 59 | 37 | 25 | 17 | 9 | 2 |

Table S3. Sample characteristics before and after GPS cleaning process

| Variables | Category | Before GPS cleaning (*N* = 629) | Final sample (*N* = 141) |
| --- | --- | --- | --- |
| GAD-7 score | N-Miss | 6 |  |
|  | Mean (SD) | 4.059 (4.226) | 4.099 (4.341) |
| Age | N-Miss | 1 |  |
|  | Mean (SD) | 45.094 (14.086) | 43.660 (14.047) |
| Sex | N-Miss | 1 |  |
|  | Male [*N* (%)] | 327 (52.1%) | 73 (51.8%) |
|  | Female [N (%)] | 301 (47.9%) | 68 (48.2%) |
| Employment | N-Miss | 1 |  |
|  | Employed [N (%)] | 449 (71.5%) | 102 (72.3%) |
|  | Unemployed [N (%)] | 179 (28.5%) | 39 (27.7%) |
| Income | N-Miss | 3 |  |
|  | Mean (SD) | 3.637 (1.262) | 3.574 (1.321) |
|  | Very_low [N(%)] | 52 (8.3%) | 15 (10.6%) |
|  | Low [N(%)] | 73 (11.7%) | 15 (10.6%) |
|  | Middle [N(%)] | 121 (19.3%) | 30 (21.3%) |
|  | High [N(%)] | 184 (29.4%) | 36 (25.5%) |
|  | Very high [N(%)] | 196 (31.3%) | 45 (31.9%) |
| Marital status | N-Miss | 1 |  |
|  | Married [N (%)] | 345 (54.9%) | 78 (55.3%) |
|  | Unmarried [N (%)] | 283 (45.1%) | 63 (44.7%) |
| Education | N-Miss | 3 |  |
|  | Low [N (%)] | 81 (12.9%) | 14 (9.9%) |
|  | Mid [N (%)] | 240 (38.3%) | 61 (43.3%) |
|  | High [N (%)] | 305 (48.7%) | 66 (46.8%) |

Table S4: Associations between the GAD-7 scores and the exposure patterns. Exposures were based on 100 m buffers and 10 min time window. The model was adjusted for age, sex, income, employment status, marital status, and educational background.

| Variables | 100 m buffer | |
| --- | --- | --- |
|  | B | *SE* |
| Cluster 2: Moderately health-threatening | -2.518 * | 1.093 |
| Cluster 3: Moderately health-supportive | -1.889 ▪ | 0.959 |
| Cluster 4: Strongly health-supportive | -1.249 | 0.951 |

* = p < 0.05; ▪ = p < 0.1


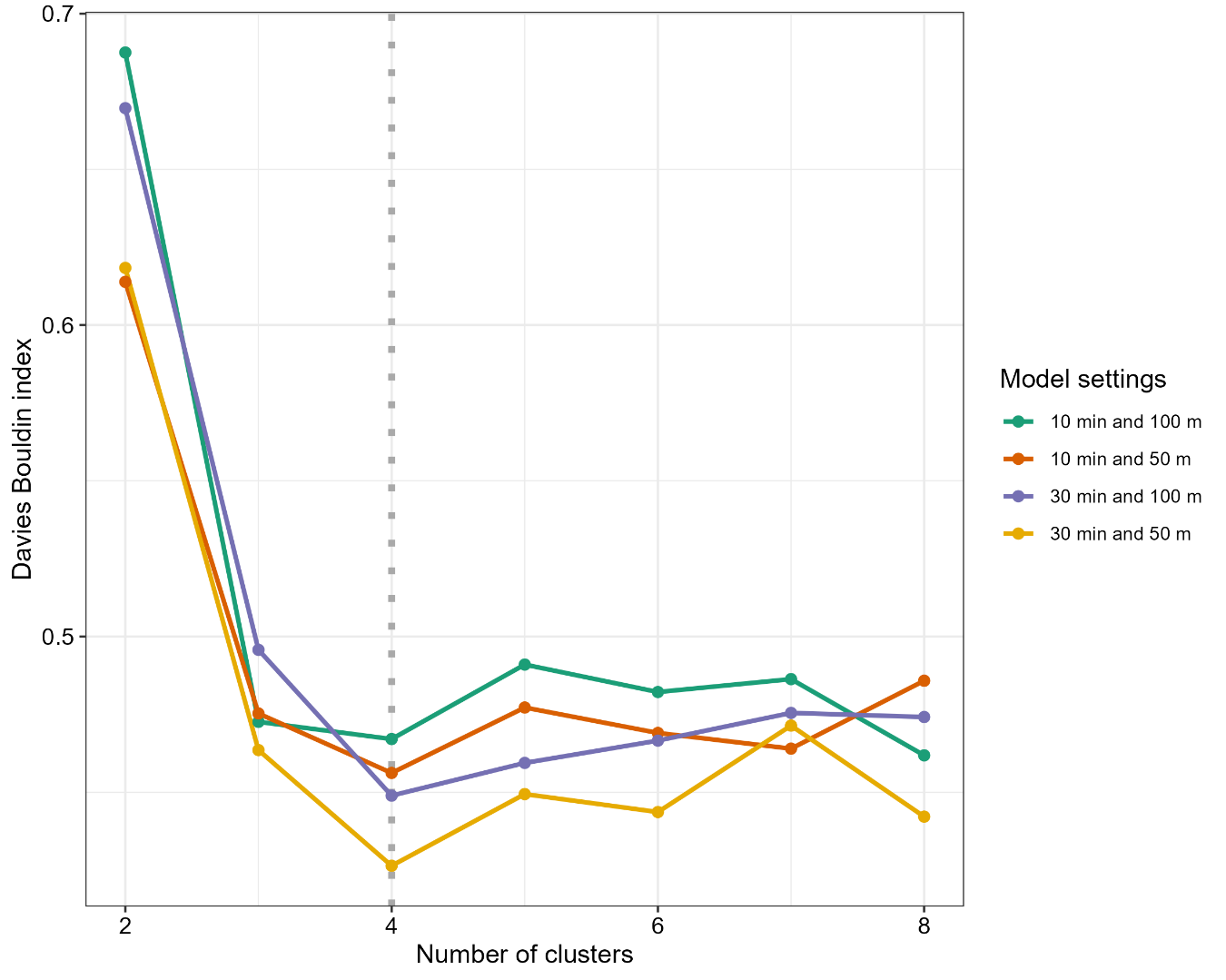


Figure S1: Davies Bouldin index for different numbers of clusters and across various model settings (i.e., time windows and buffer sizes).


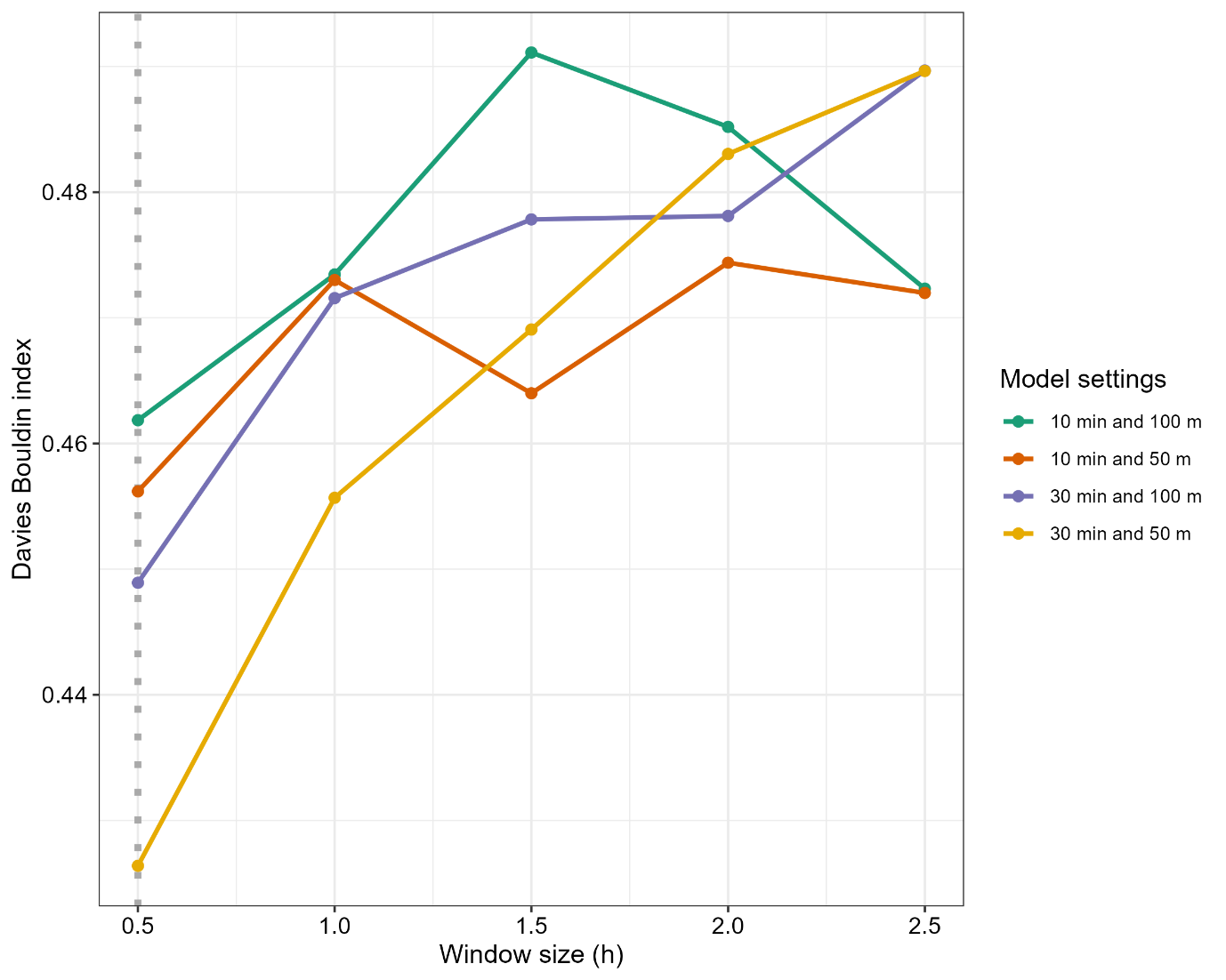


Figure S2: Davies Bouldin index for different window size of constraints and across various model settings (i.e., time windows and buffer sizes).


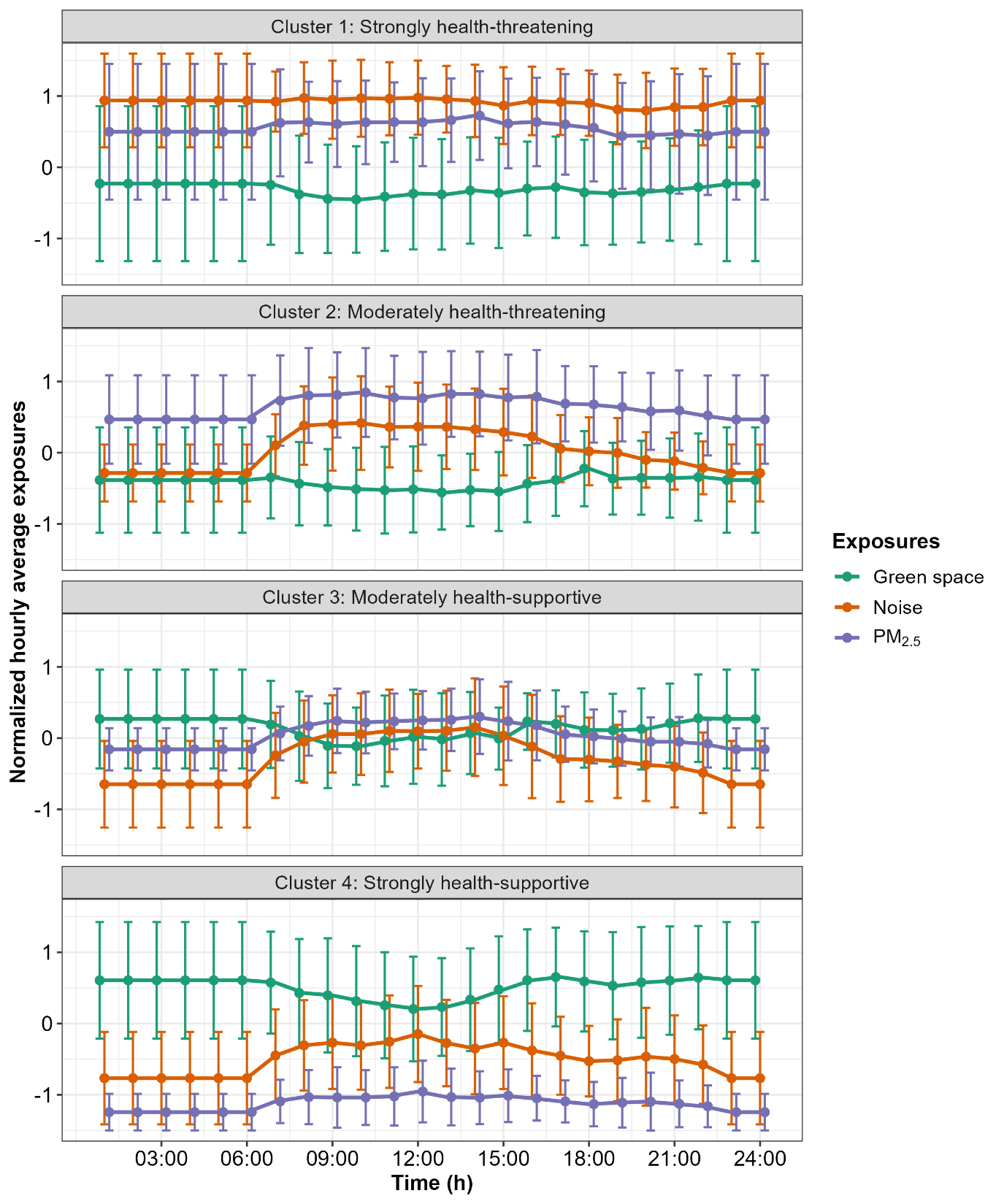


Figure S3: Sequential environmental exposure patterns of the four clusters based on 50 m buffers and 10-min time windows. The data partition was conducted with multivariate time series clustering and constrained dynamic time warping.


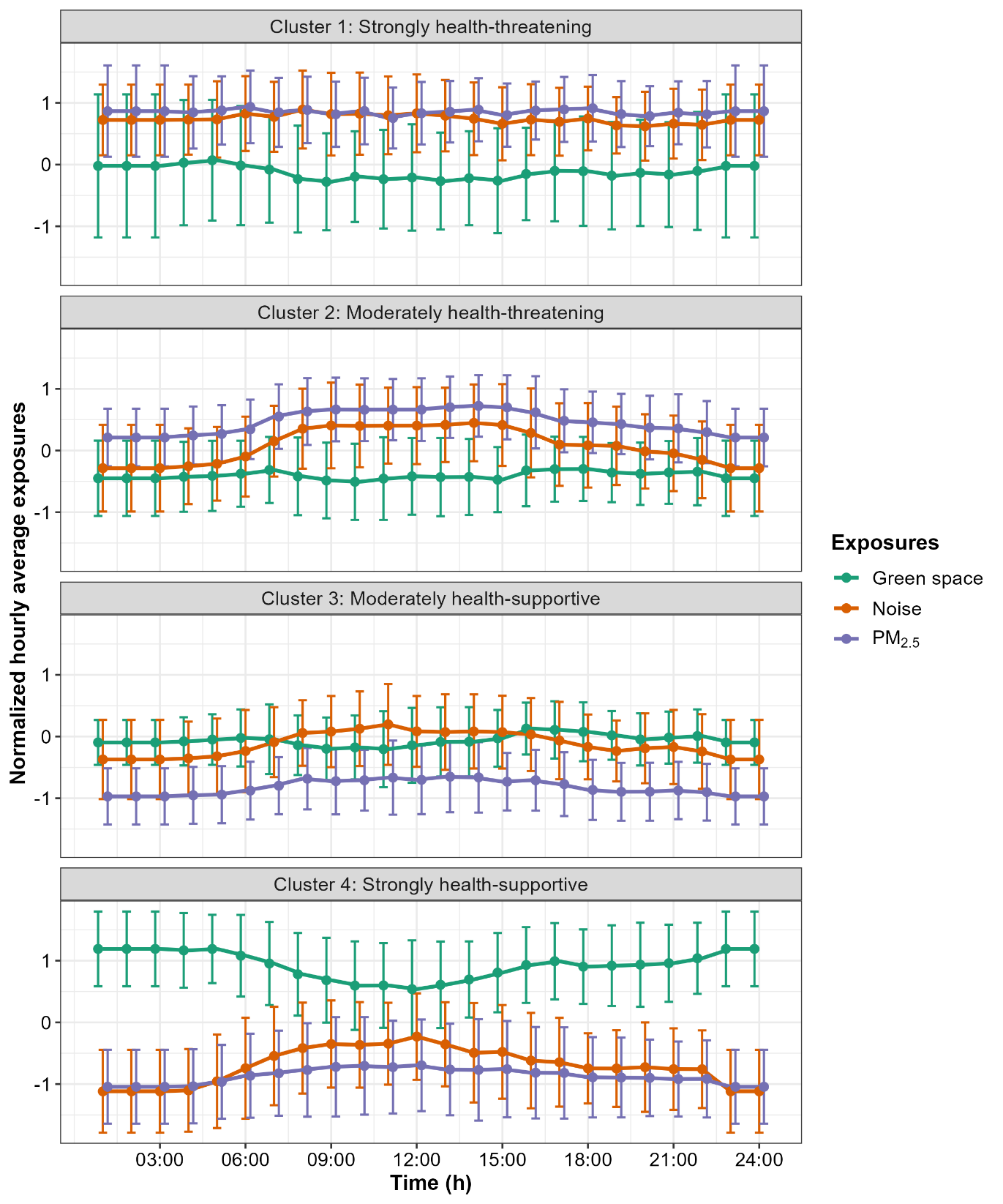


Figure S4: Sequential environmental exposure patterns of the four clusters based on 100 m buffers and 30-min time windows. The data partition was conducted with multivariate time series clustering and constrained dynamic time warping.


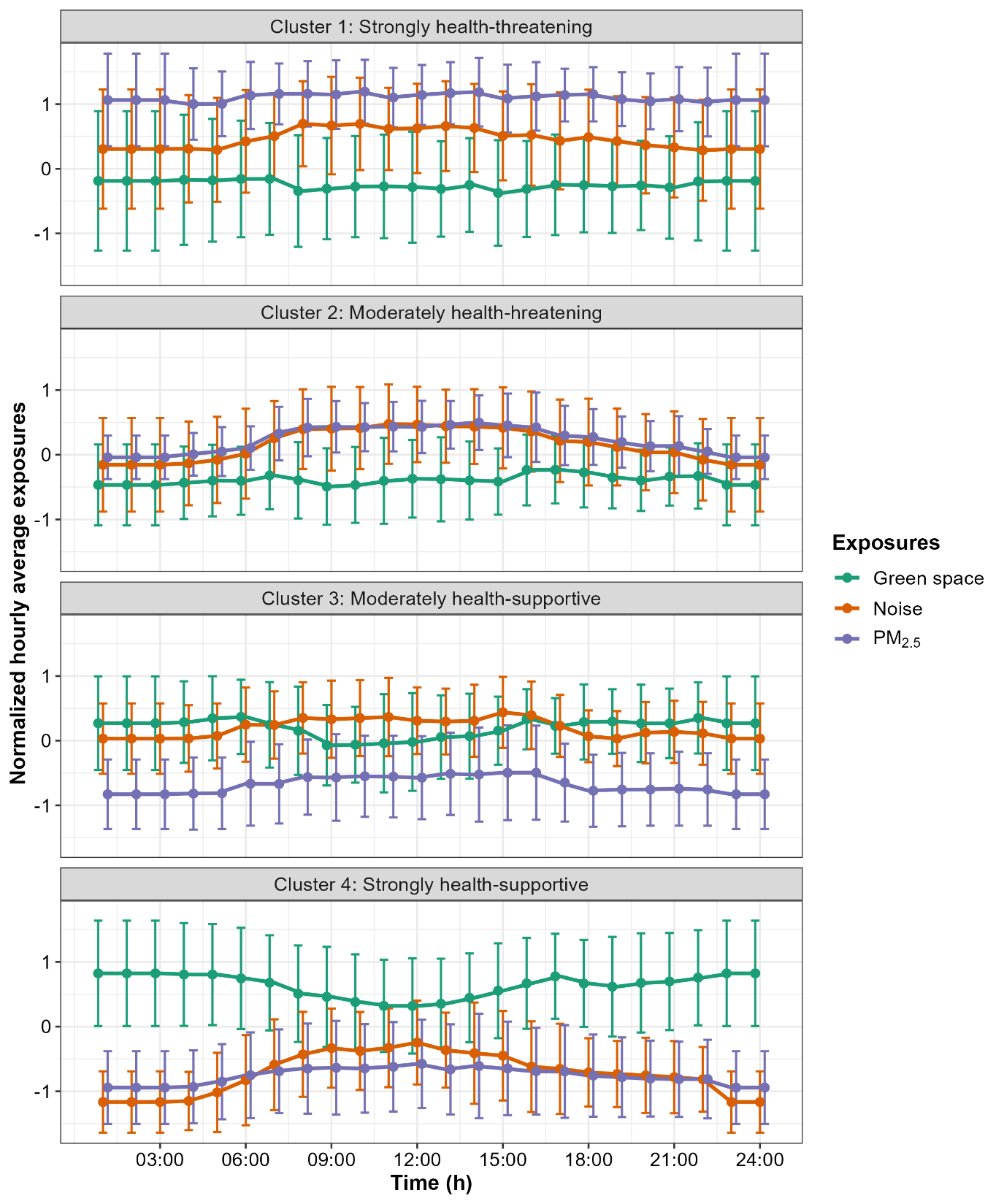


Figure S5: Sequential environmental exposure patterns of the four clusters based on 50 m buffers and 30-min time windows. The data partition was conducted with multivariate time series clustering and constrained dynamic time warping.


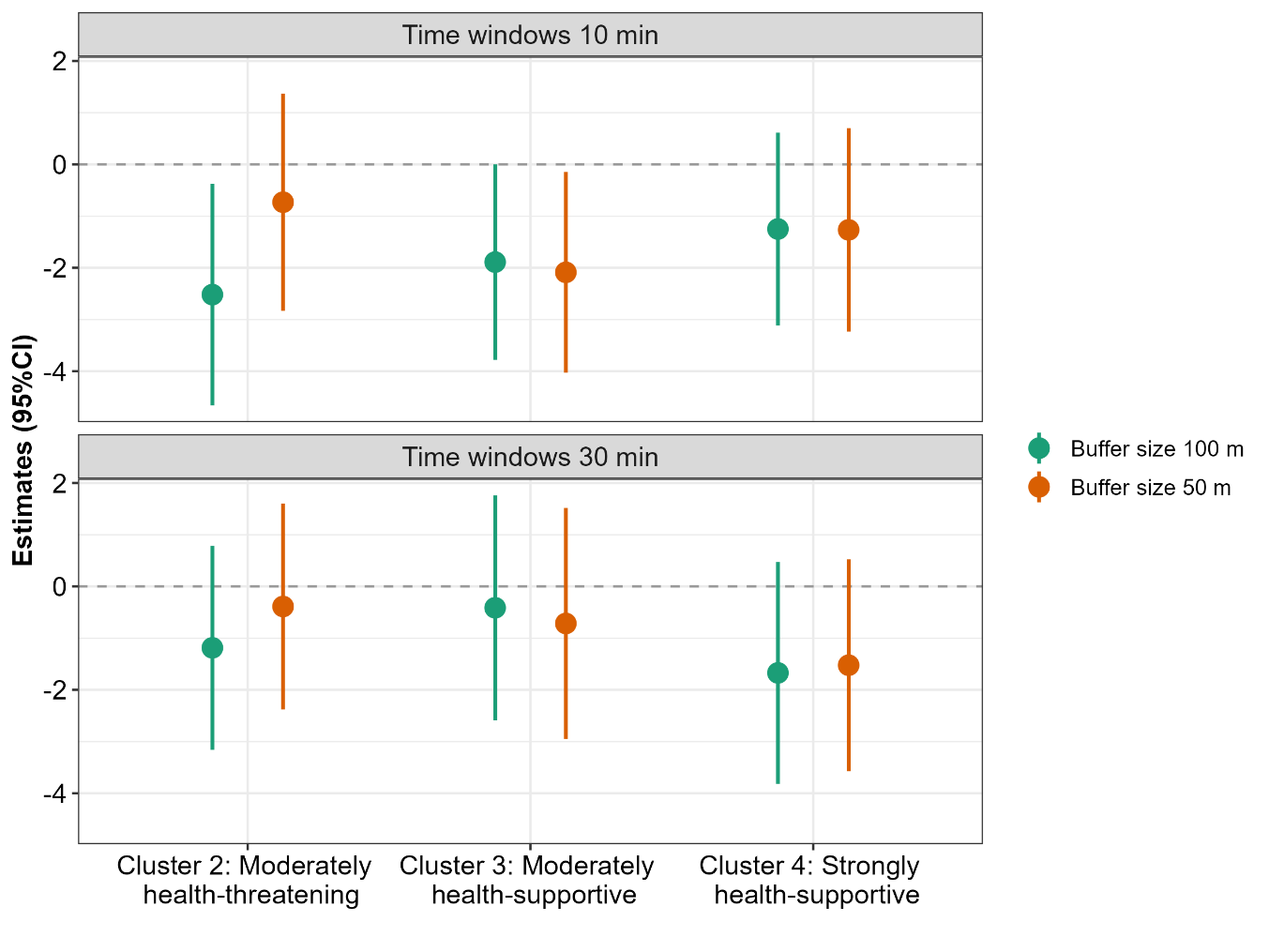


Figure S6: Regression coefficients for the associations between the GAD-7 scores and the exposure patterns across different buffers (50 m and 100 m) and time windows (10 min and 30 min). Cluster 1 (“strongly health-threatening”) served as the reference category. The model was adjusted for age, sex, income, employment status, marital status, and educational background.


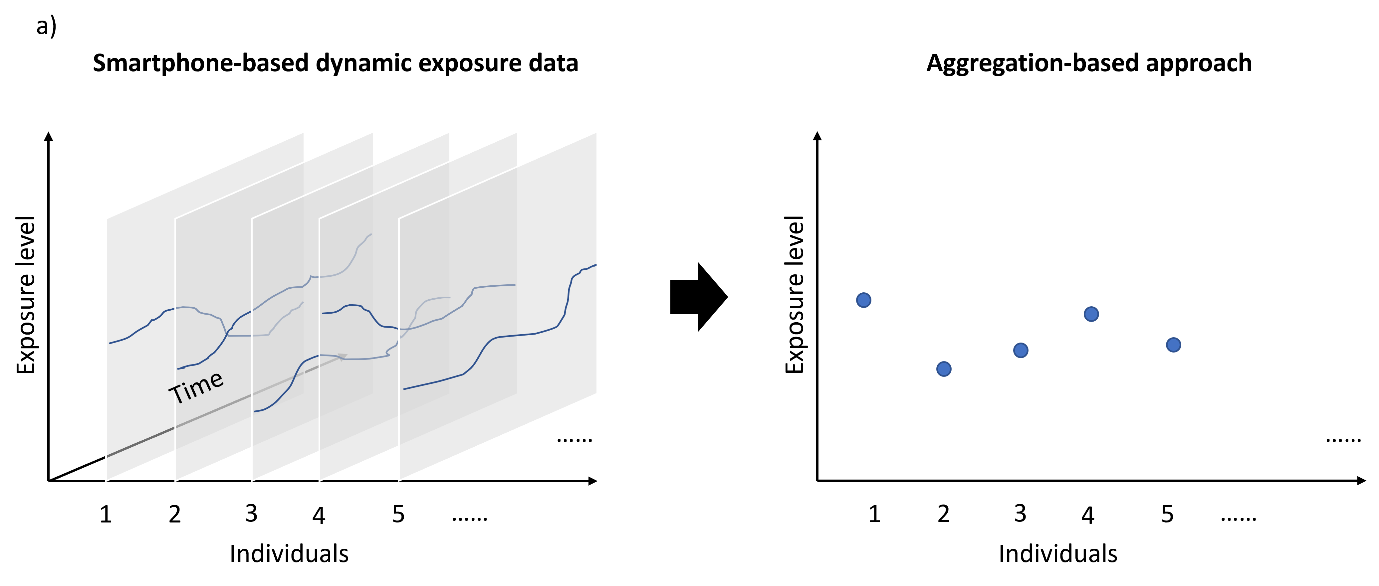


Figure S7: Preprocessing of mobility-based exposure data using aggregation-based approach.


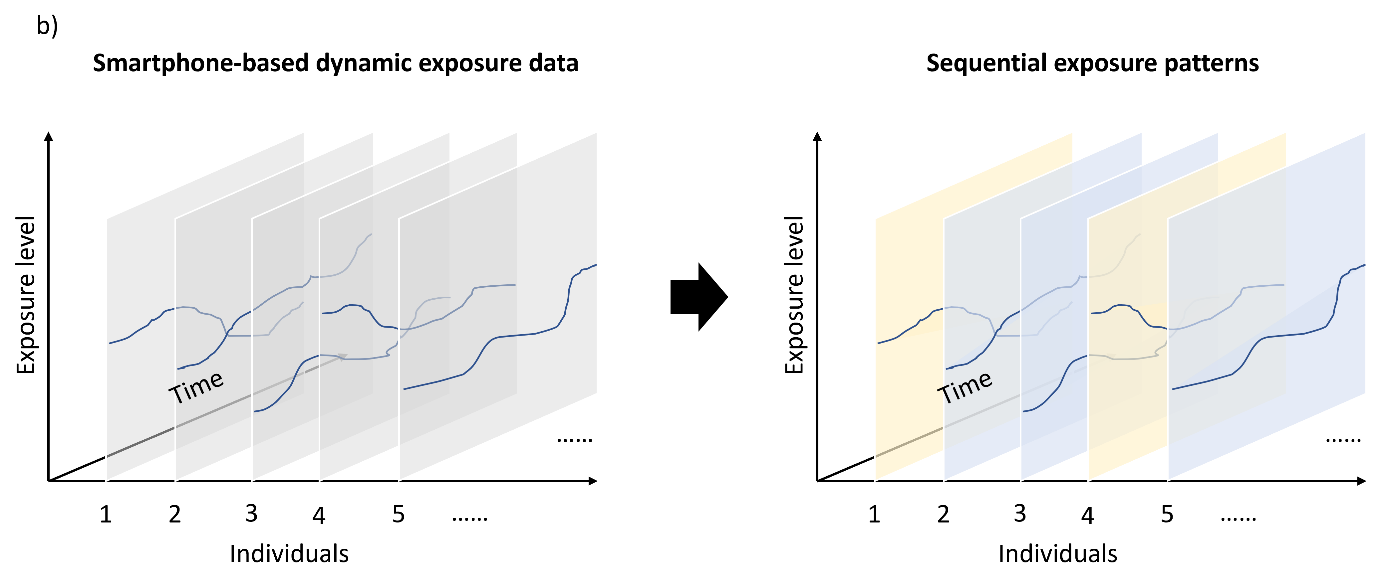


Figure S8: Preprocessing of mobility-based exposure data in our study using the time-series approach. Individuals of the same color were assigned in the same cluster.
